# Global analysis of bovine milk protein variants using multi-breed DNA sequence data

**DOI:** 10.1186/s12864-026-12836-2

**Published:** 2026-04-10

**Authors:** Ying Liu, Etske Bijl, Junxin Gao, Rayner Gonzalez-Prendes, Martien A. M. Groenen, Juha Kantanen, Catarina Ginja, Nasser Ghanem, Donald Rugira Kugonza, Mahlako Makgahlela, Henk Bovenhuis, Richard P. M. A. Crooijmans

**Affiliations:** 1 Animal Breeding and Genomics, Wageningen University and Research, Wageningen, 6700 AH The Netherlands; 2 Food Quality and Design Group, Wageningen University and Research, Wageningen, 6700 AA The Netherlands; 3 Natural Resources Institute Finland, Jokioinen, Finland; 4 Faculty of Veterinary Medicine, CIISA, University of Lisbon, Lisbon, Portugal; 5 Animal Production Department, Faculty of Agriculture, Cairo University, Giza, Egypt; 6 Department of Agricultural Production, College of Agricultural and Environmental Sciences, Makerere University, Kampala, Uganda; 7 Agricultural Research CouncilAnimal Production Institute, Irene, South Africa; 8 Department of Animal, Wildlife and Grassland Sciences, University of the Free State, Bloemfontein, South Africa

**Keywords:** Milk protein, Genome sequencing, Genetic variation, Global cattle breeds

## Abstract

**Background:**

To date, 63 variants of six major bovine milk proteins (α_S1_-CN, β-CN, α_S2_-CN, κ-CN, α-LA, and β-LG) have been described. These variants are caused by changes in the amino acid sequence of the mature protein, primarily resulting from missense variations in the exons of genes or splice sites. Several of these variants are known to be associated with milk production traits, cheese-processing properties, and the nutritional value of milk. In the past, milk protein variants have been identified in a limited number of breeds, especially in dairy cattle. The objective of this study was to investigate variation in milk proteins in a large number of cattle breeds based on whole genome sequencing data.

**Results:**

We investigated variants of the six major milk proteins in 3,824 cattle representing 113 breeds with wide geographical distribution using whole genome sequencing data. 59 missense variants of milk protein genes that can alter the amino acid sequence of the mature protein were detected. Notably, 10 out of 11 missense variants in *CSN3* were located within the region coding for the glyco-macropeptide, whereas the para-κ-casein region involved in micelle stabilization remained highly conserved with only one variant detected, suggesting functional constraint in this region. A total of 121 milk protein variants were identified based on different combinations of the 59 missense variants, of which 35 had been described previously. We detected 86 novel variants that had not been reported previously. These protein variants were likely missed in earlier studies due to technical limitations or the use of limited number of animals or breeds.

**Conclusion:**

This study provides a comprehensive overview of milk protein diversity across global cattle breeds, offering valuable insights for improving milk quality and properties, guiding selective breeding and prioritizing variants for future functional investigation in the dairy sector.

**Supplementary Information:**

The online version contains supplementary material available at 10.1186/s12864-026-12836-2.

## Background

Cow milk proteins provide suckling calves with essential nutrients, including amino acids, calcium, phosphorus and potentially bioactive peptides. Moreover, milk proteins are the primary macromolecules responsible for the production of many dairy products, such as cheese and yoghurt. About 80% of the total bovine milk protein fraction is composed of four caseins (α_S1_-CN, α_S2_-CN, β-CN, and κ-CN) [1]. The remaining approximately 20% of the bovine milk proteins consists of two major whey proteins α-lactalbumin (α-LA) and β-lactoglobulin (β-LG), which are present in the serum phase of milk. These six major milk proteins are encoded by six genes. The 4 major casein genes *CSN1S1* (α_S1_-CN), *CSN2* (β-CN), *CSN1S2* (α_S2_-CN), and *CSN3* (κ-CN) which are located within a 250-kb genomic region on Bos taurus autosome 6 (BTA 6) (between 85.4 and 85.7 Mb on genome assembly ARS-UCD1.2). The genes coding for the main whey proteins, *LALBA* (α-LA) and *PAEP* (β-LG) are located on BTA5 and BTA11, respectively [2].

Bovine milk protein genes have been extensively investigated and characterized and all genes have been shown to be polymorphic [3]. Until now, a total of 63 protein variants have been reported [3–6]. Milk protein variants are caused by missense variations or splice sites variations, resulting in amino acid changes in the mature protein. Additional DNA variations occur in the regulatory regions of genes which can modulate (increase/decrease) the gene expression [7, 8]. Some milk protein polymorphisms have been found to lead to quantitative differences in protein composition or affect post-translational modification (PTM) [9]. Both could influence the nutritional value and technological properties of milk, which might affect the quality of dairy products [3, 10]. Thus there is a great deal of scientific interest in the identification of milk protein variants in cattle breeds.

Cattle breeds with different production characteristics have been shaped by divergent selective breeding histories. Dairy breeds have been intensively selected for high milk yield and composition to meet the demands of dairy production, whereas beef cattle have been selected mainly for growth and carcass traits. In contrast to dairy systems, where calves are separated early and fed milk replacer, calves in the beef production systems usually remain with their dams until weaning. As a result, maternal milk composition directly affects calf growth and weaning weight, which is an important breeding goal trait in beef cattle [11, 12]. κ-casein plays a critical role in milk coagulation, a process that influences the digestion rate and nutrient utilization in suckling calves. Variants that reduce renneting efficiency, such as κ-casein A and E, may negatively affect digestion and calf performance because milk passes through the abomasum too rapidly for an efficient extraction of nutrients [13]. We therefore hypothesize that these variants are under stronger purifying selection in beef than in dairy breeds.

In previous studies, two general approaches have been used to identify protein variants: separation of proteins and the DNA sequence analysis. Protein separation approaches including electrophoretic, chromatography- and mass spectrometry-based protein fractionation techniques have mainly been used for dairy cattle breeds where milk is readily available. However, these techniques separate protein variants in milk relying on differences in physicochemical characteristics such as charge, isoelectric point, hydrophobicity or molecular weight [14–17] but may fail to detect all variants. Most beef and indigenous breeds have been poorly studied on milk composition, as their main purpose is beef or draft rather than dairy products. DNA sequence analysis provides access to genomic data, which facilitate the accurate identification of milk protein variants in more variety of breeds for which milk samples are unavailable. With the development of genomic sequencing technologies, bovine whole genome sequencing (WGS) providing high quality DNA sequence data have been applied to characterize both known and novel milk protein variants accurately, while studies have so far been limited to a few breeds [4, 18, 19].

In the present study we utilize WGS data from global cattle breeds, including those from the 1000 Bull Genomes Project and the OPTIBOV Project, to give a comprehensive overview on variants of the six major milk proteins. The 1000 Bull Genomes Project (Run7.0) is a collection of WGS data from more than 3,000 individuals capturing a significant proportion of the world’s cattle diversity, ranging from commercial breeds selected for high milk or meat production in temperate environments to numerous local breeds adapted to harsh conditions [20]. The OPTIBOV Project includes whole-genome sequence data of local bovine breeds from six countries, adapted to diverse environments in Europe and Africa (https://www.optibov.org/). This study provides a comprehensive diversity assessment of the major milk protein variants of global cattle breeds which may provide insights into positive selection on milk properties, breeding strategies and dairy product quality improvement.

## Results

### DNA sequence variations

The analysis of the six major bovine milk protein genes including the 2,000 bp flanking regions identified 3,404 genetic variants relative to the bovine reference sequence. Of these variants, 1,744 were known and 1,660 were novel (Supplementary Table S2). A total of 463 variants were located in the upstream and 579 in the downstream regions. Within the six milk protein genes, 2,362 genetic variants were identified, with polymorphic sites accounting for 3.56% of the total base pairs. Most variants within the milk protein genes were located in intronic regions (89.9%), slightly lower than the genome-wide average (95.8%) (Table 1). Variants located in untranslated regions (UTRs) accounted for 5.75%, which is higher than the genome-wide average (1.05%). Missense variants accounted for 2.8% of all polymorphisms, nearly twice the whole bovine genome proportion (1.6%).

**Table 1:** Percentage of the polymorphisms in milk protein genes and the whole bovine genome annotated to the different DNA variant types

Variant types	[%] milk protein genes	[%] whole bovine genome^a^
5 prime UTR	3.04%	0.2%
Missense	2.84%	1.57%
Synonymous	1.53%	1.52%
Intron	89.89%	95.84%
3 prime UTR	2.71%	0.85%

^a^whole bovine genome: exclude intergenic region

### Missense variants

**Fig. 1:**
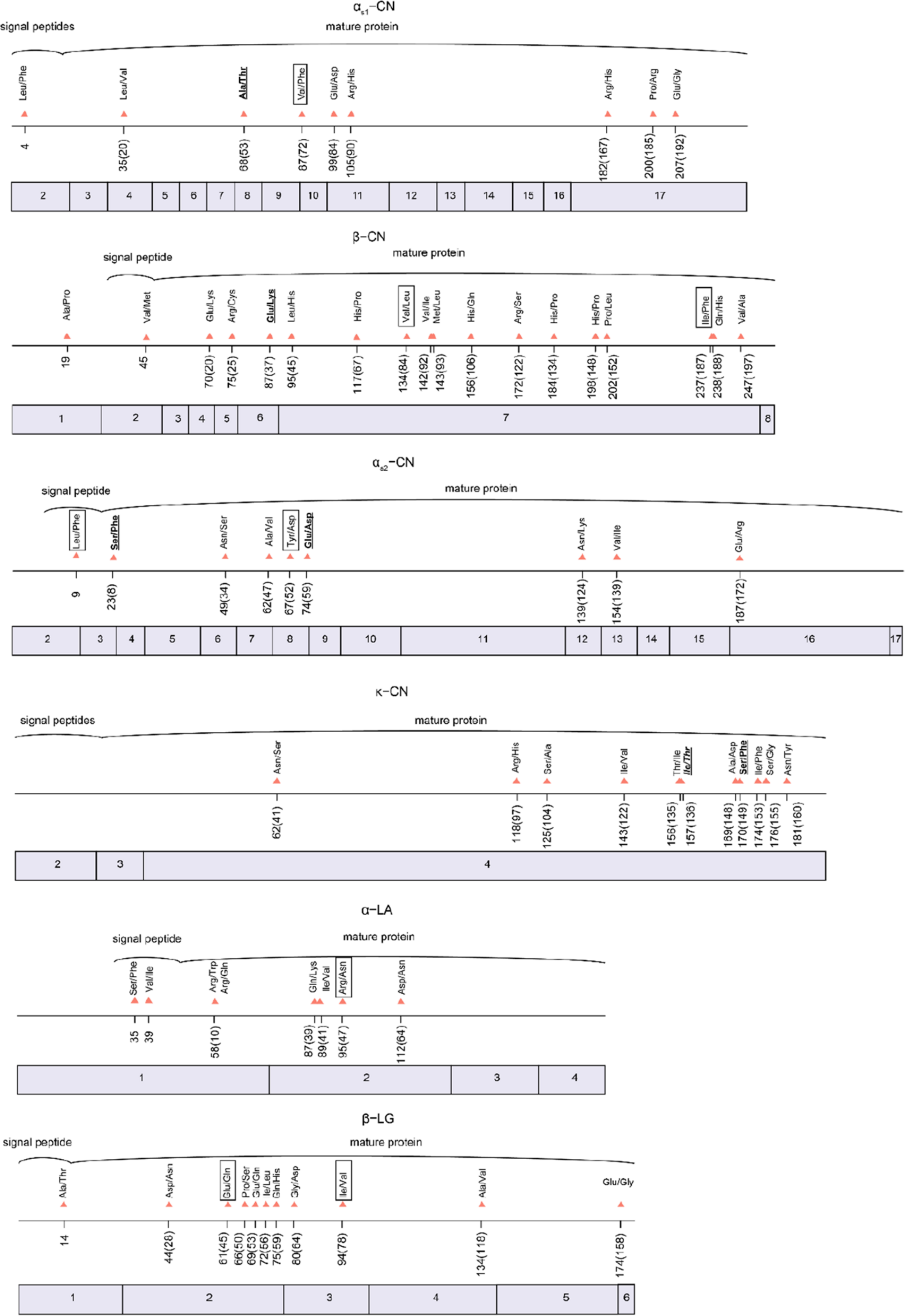
Amino acid variations in the 6 major milk protein genes. Exon number and amino acid variations are numbered according to the reference protein sequence from the ARS-UCD1.2 assembly. Positions in the mature protein are given in parentheses. Protein variants coded by the reference genome sequence are: *CSN1S1*: α_S1_-CN B; *CSN2*: β-CN B; *CSN1S2*: α_S2_-CN A; *CSN3*: κ-CN B; *LALBA*: α-LA B; *PAEP*: β-LG B. Amino acid variations related to phosphorylation sites are in bold and underlined; glycosylation-related variants are in bold italic and underlined. The rarest variants (observed only twice) are in text boxes

A total of 66 missense variants were identified within the coding regions of the six major milk protein genes. Detailed information including the amino acid changes, location, SIFT (Sorting Intolerant From Tolerant) score and allele frequencies of missense variants across breeds and the total population was provided in Supplementary Table S4. The missense variants for the six milk protein genes and their corresponding positions within the protein are illustrated in Fig. 1. Among the identified variants, eight missense variants were the most rare and each detected in only two individuals.

Of the in total 66 missense variations, 7 could not alter the mature protein. One missense variant, which initially was annotated to the first exon of *CSN2*, actually does not code for the β-CN protein. This suggests a mis-annotation in the reference genome. The other 6 variants (one each in *CSN1S1*, *CSN2*, *CSN1S2*, *PAEP*, and two in *LALBA*) that cannot alter the mature protein are located in the region coding for the signal peptide, and these have no effect on the mature protein. The remaining 59 missense variants change the amino acid sequence of the mature proteins.

The distribution of missense variants varied among genes (Fig. 1). In *CSN1S1*, eight variants were identified in exons 2, 4, 8, 10, 11 and 17, and exons 10, 11 and 17 appear to accumulate more variants. Sixteen missense variants in *CSN2* were distributed across the gene. In *CSN1S2*, eight missense variants were located in exons 2, 3, 6, 7, 8, 12, 13, and 16. *CSN3* displayed a strong localization pattern, with all eleven variants confined to exon 4. In *LALBA*, six missense variants were detected in exons 1 and 2, whereas no missense mutations were detected in exons 3 and 4. In *PAEP*, ten missense variants were found in exons 1, 2, 3, 4, and 6.

### Milk protein variant detection

**Fig. 2:**
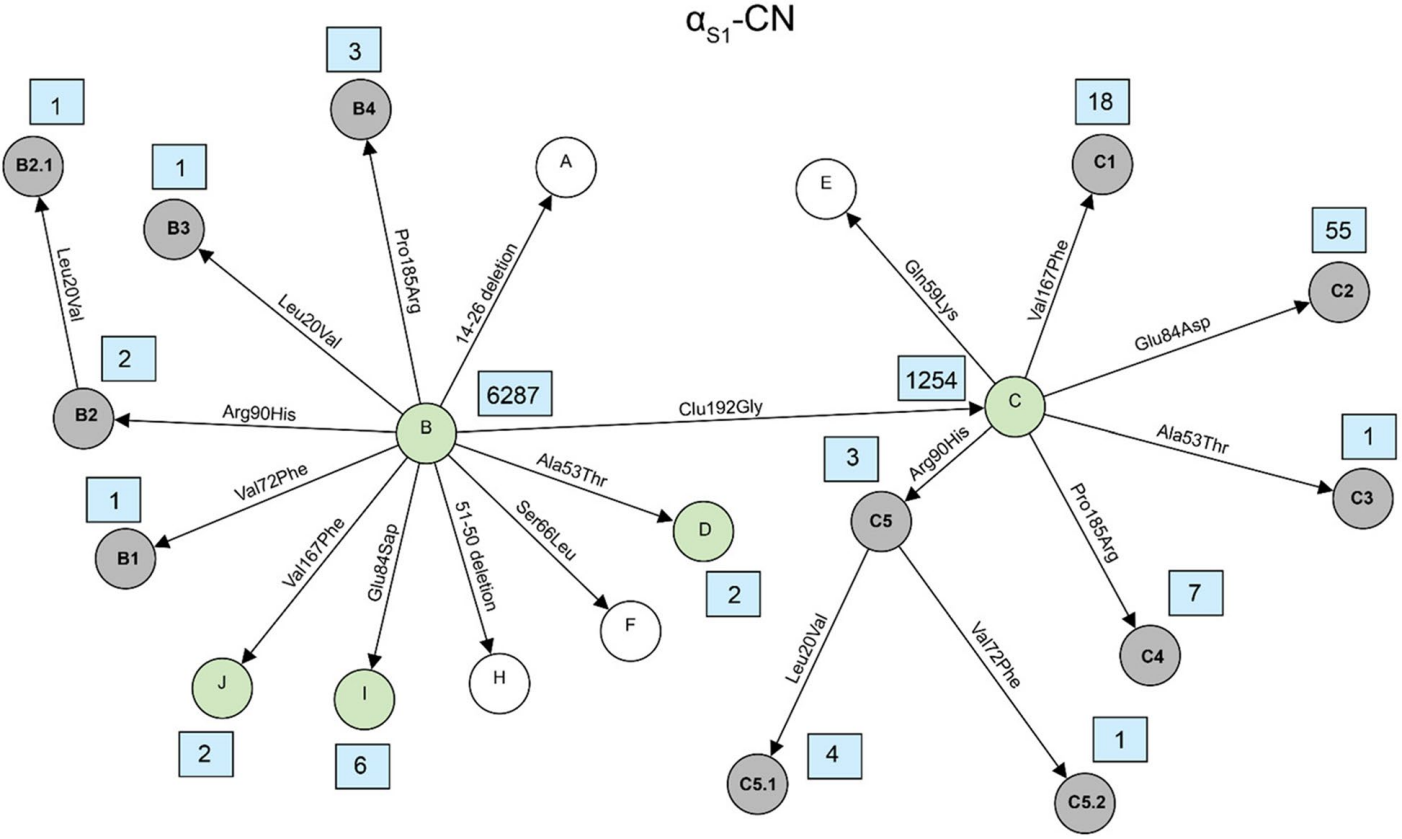
Network diagram of amino acid changes among protein variants of α_S1_-CN. Known variants which are detected in this study are shown in green circles, known variants but not detected in this study are shown in white circles and novel variants described for the first time in this study are shown in grey circles. Occurrences of each variant, which are defined here as the number of times each variant was observed in the dataset, are indicated in a blue rectangle next to its circle

**Fig. 3:**
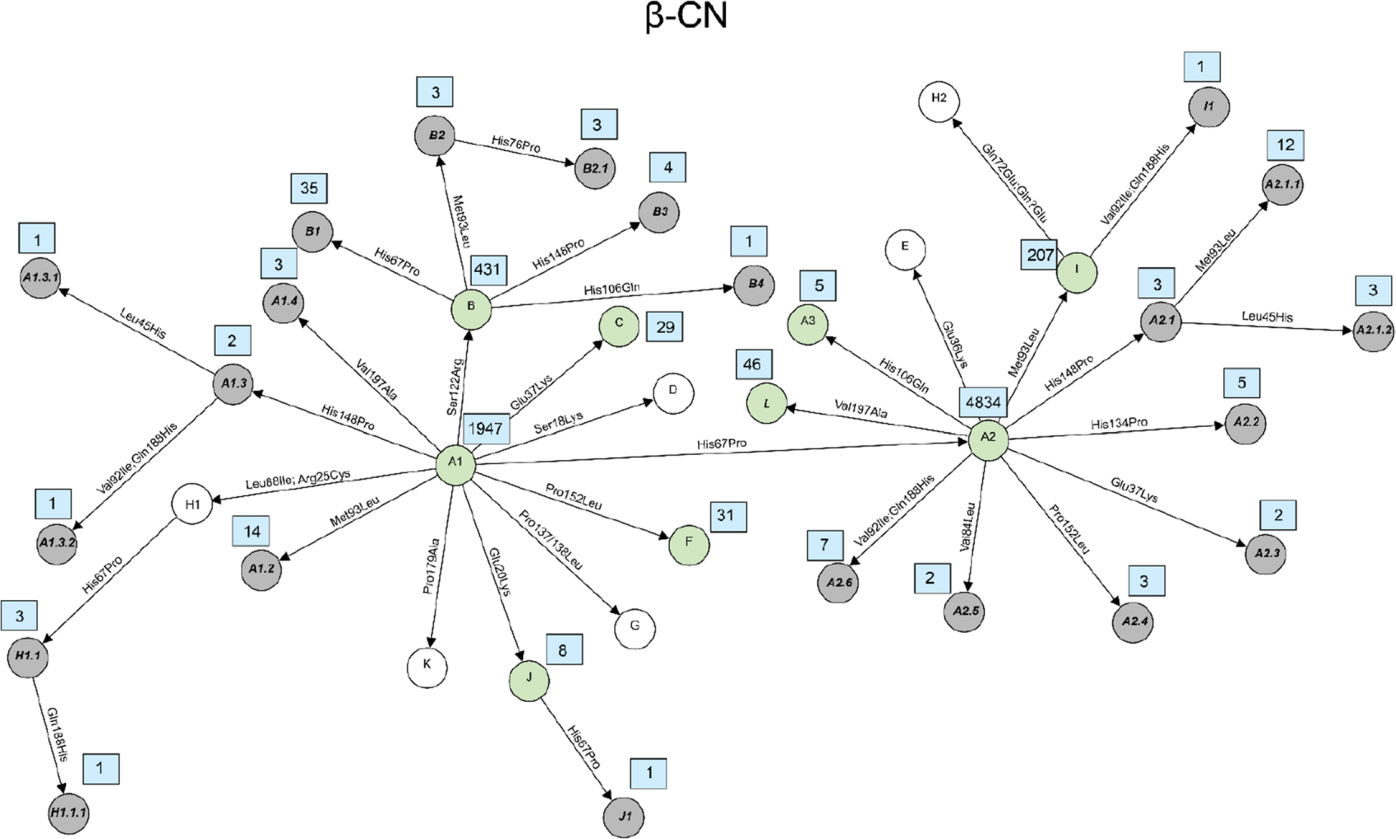
Network diagram of amino acid changes among protein variants of β-CN. Known variants which are detected in this study are shown in green circles, known variants but not detected in this study are shown in white circles and novel variants described for the first time in this study are shown in grey circles. Occurrences of each variant, which are defined here as the number of times each variant was observed in the dataset, are indicated in a blue rectangle next to its circle

**Fig. 4:**
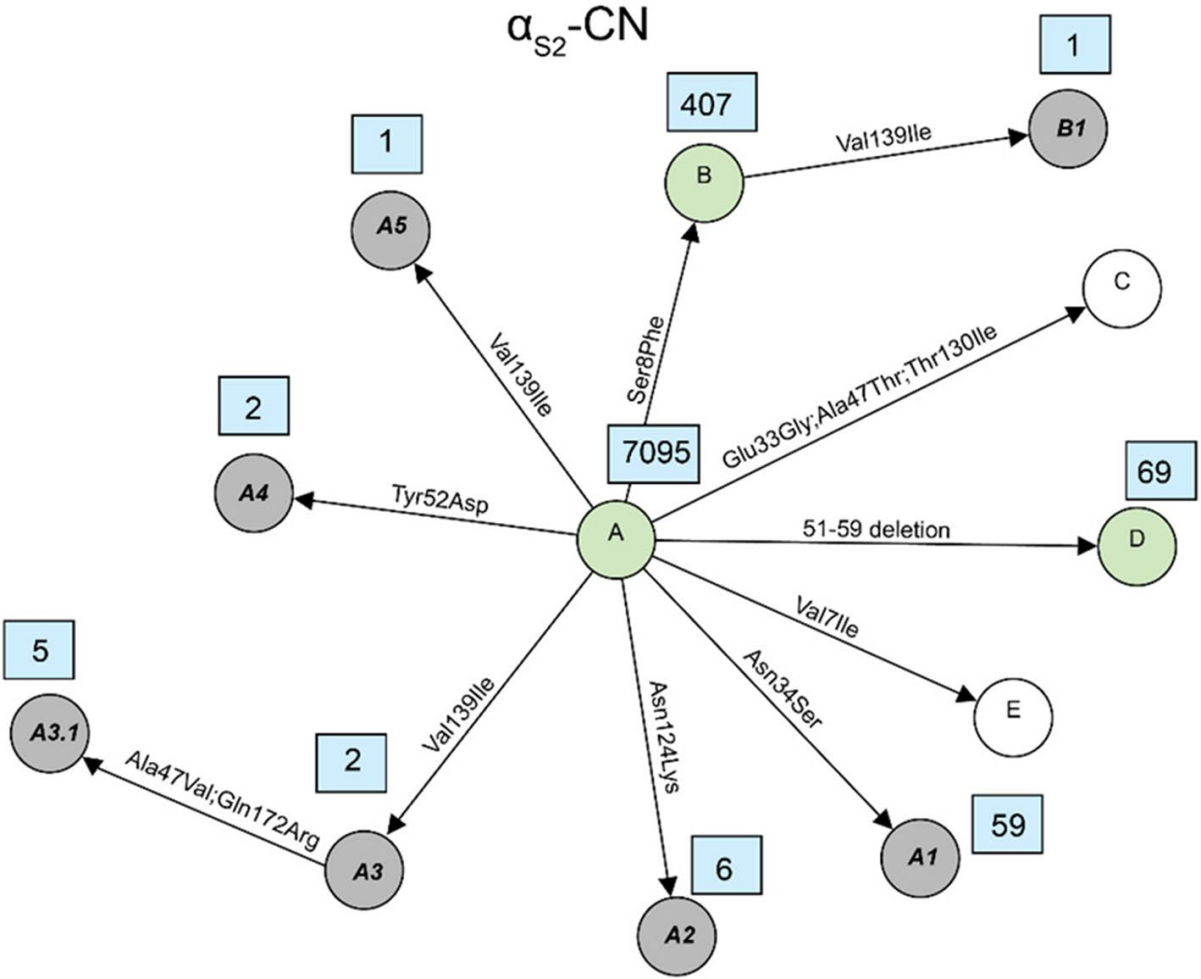
Network diagram of amino acid changes among protein variants of α_S2_-CN. Known variants which are detected in this study are shown in green circles, known variants but not detected in this study are shown in white circles and novel variants described for the first time in this study are shown in grey circles. Occurrences of each variant, which are defined here as the number of times each variant was observed in the dataset, are indicated in a blue rectangle next to its circle

**Fig. 5:**
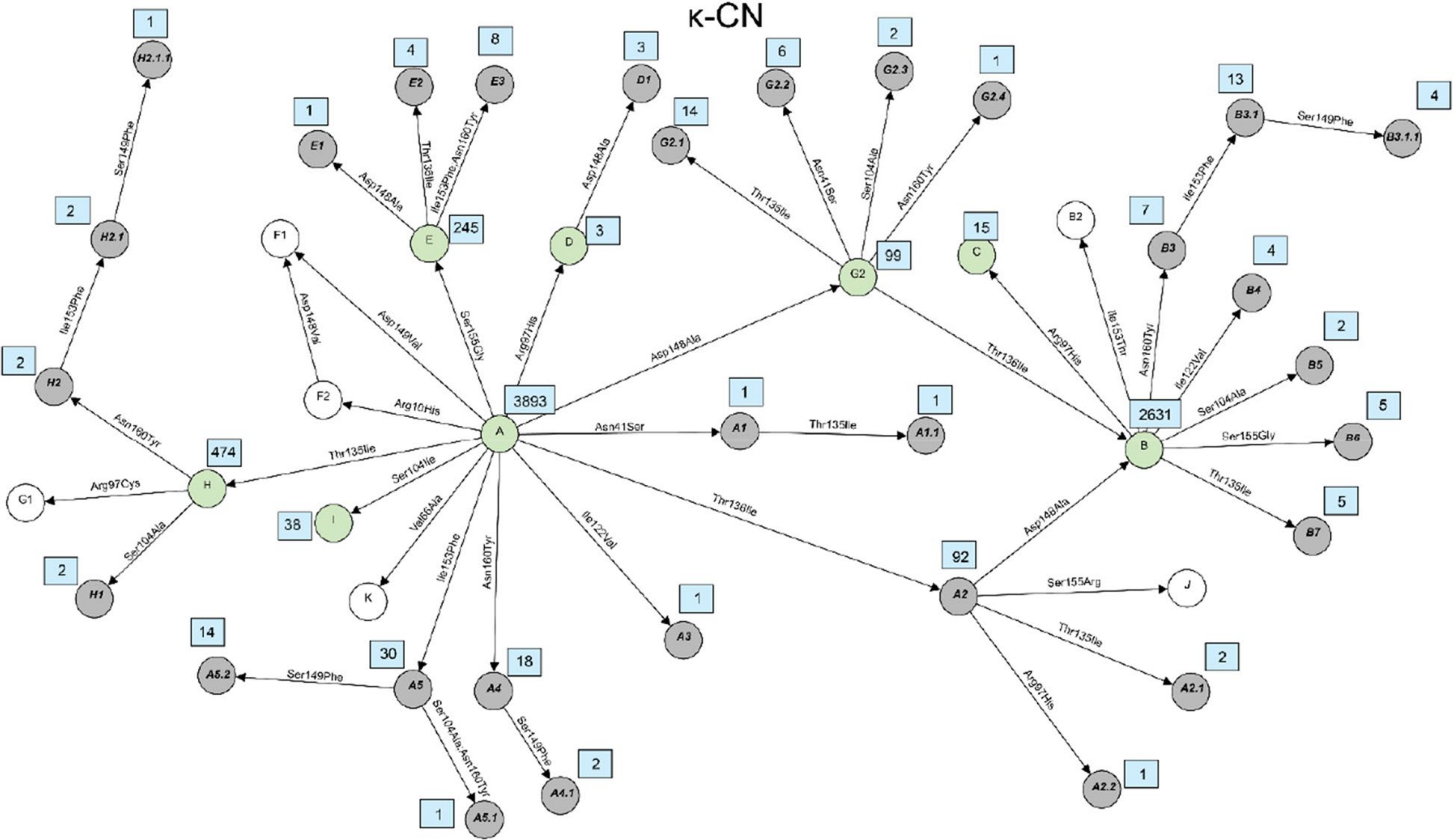
Network diagram of amino acid changes among protein variants of κ-CN. Known variants which are detected in this study are shown in green circles, known variants but not detected in this study are shown in white circles and novel variants described for the first time in this study are shown in grey circles. Occurrences of each variant, which are defined here as the number of times each variant was observed in the dataset, are indicated in a blue rectangle next to its circle

**Fig. 6:**
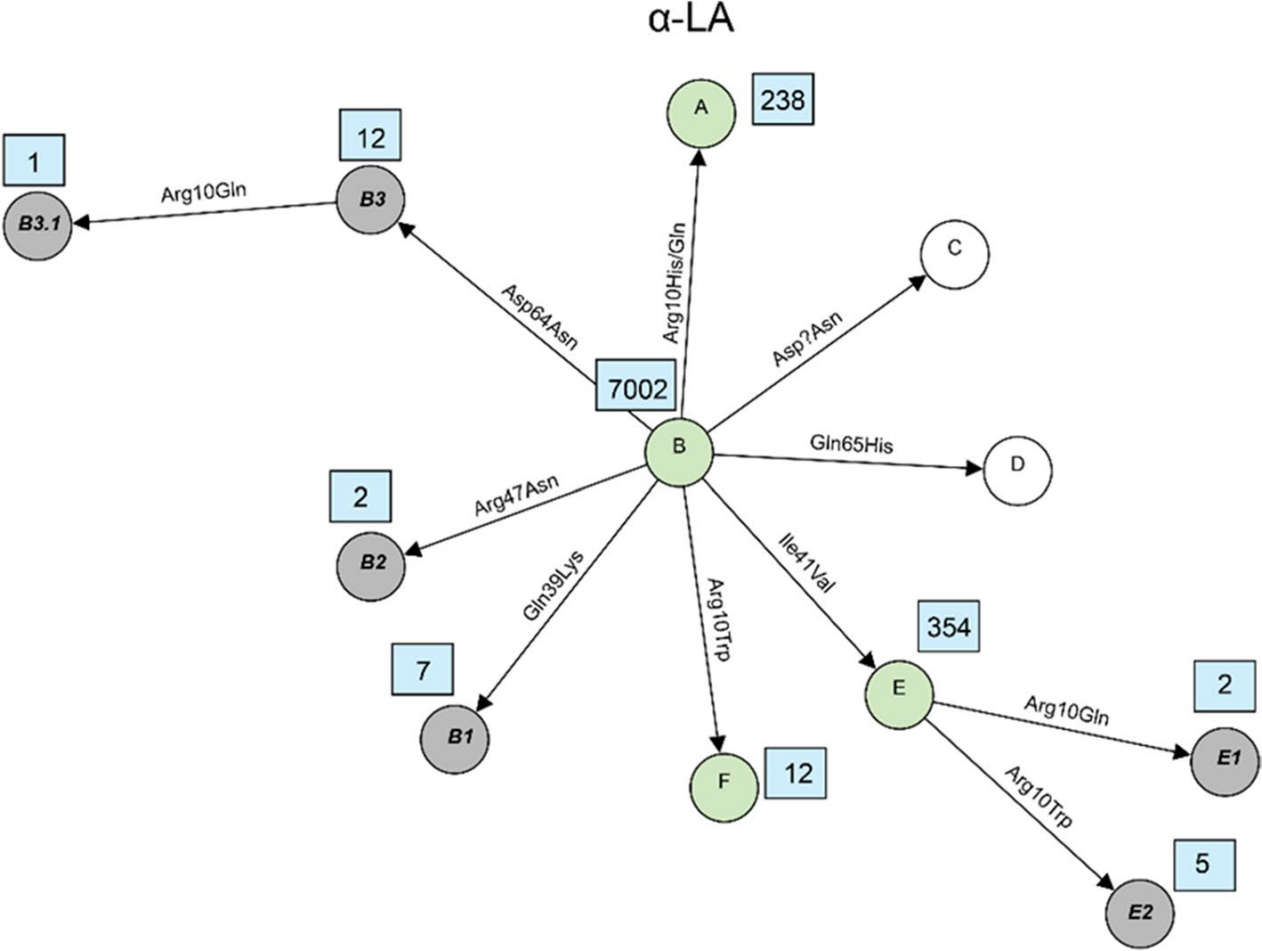
Network diagram of amino acid changes among protein variants of α-LA. Known variants which are detected in this study are shown in green circles, known variants but not detected in this study are shown in white circles and novel variants described for the first time in this study are shown in grey circles. Occurrences of each variant, which are defined here as the number of times each variant was observed in the dataset, are indicated in a blue rectangle next to its circle

**Fig. 7:**
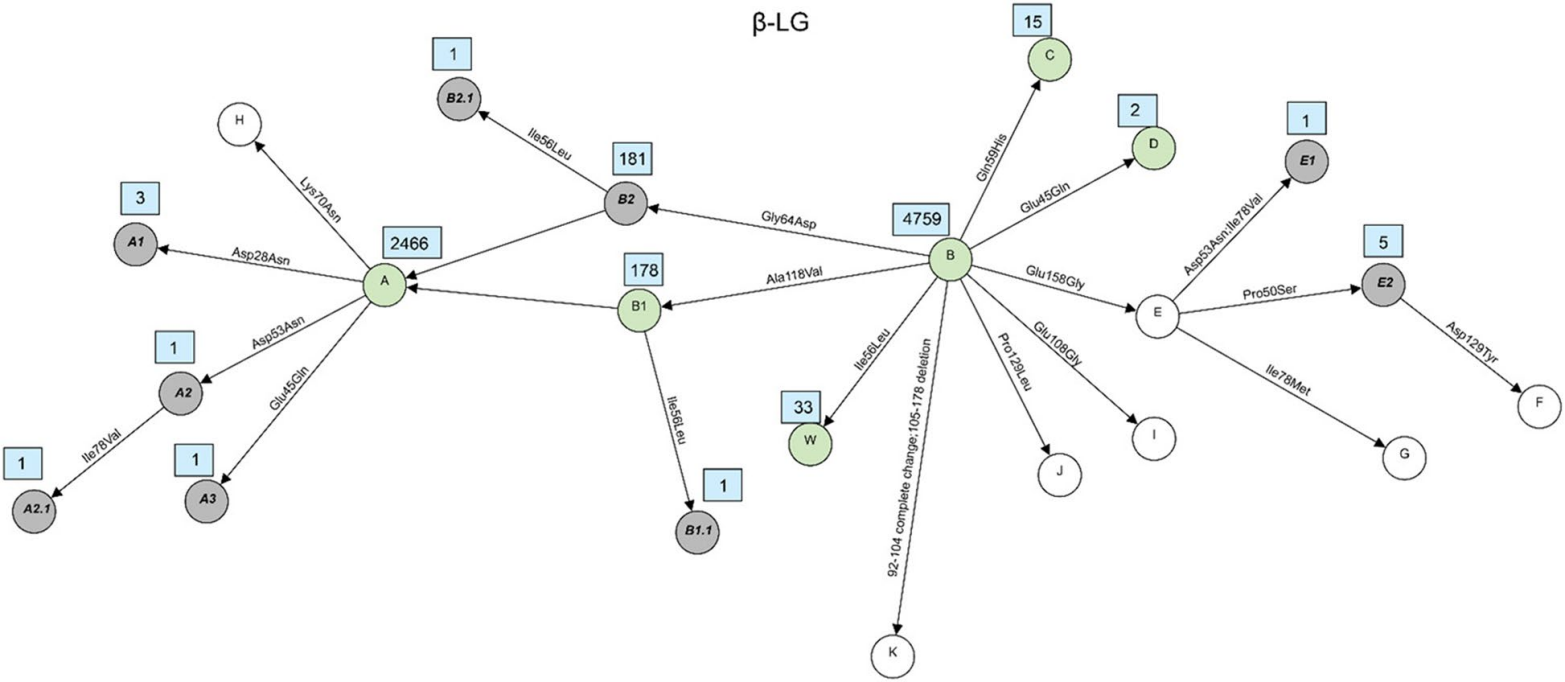
Network diagram of amino acid changes among protein variants of β-LG. Known variants which are detected in this study are shown in green circles, known variants but not detected in this study are shown in white circles and novel variants described for the first time in this study are shown in grey circles. Occurrences of each variant, which are defined here as the number of times each variant was observed in the dataset, are indicated in a blue rectangle next to its circle

The milk protein variants can be the result of combinations of two or more sequence variants and therefore were determined based on haplotypes that were constructed for each of the six milk protein genes. A total of 121 milk protein variants were identified based on different combinations of the 59 missense variants. Violin plots showing the distribution of sequencing read depth at missense variant sites were generated for each gene (Supplementary Figure S1-S6). The plots revealed sufficient coverage across sites with mean read depths above 8, indicating that protein variant identification was based on adequately covered sequencing data. In the past, the established systematic nomenclature in which almost all previously newly identified variants were named sequentially in alphabetical order. However, as the number of newly identified variants increases, existing nomenclature system becomes difficult to scale and provides limited traceability of relationships among closely related variants. Therefore, in this study, we proposed a hierarchical nomenclature system for the novel variants that represents an extension of the already known variant nomenclature. We suggest preliminary names for newly identified milk protein variants based on their origin and relationship. The name of the new variant is formed by adding a dot and a numerical digit to the base variant’s name, reflecting successive layers of amino acid substitutions. For example, variants derived from a base variant named A1 are labeled A1.1, A1.2, A1.3, and so on. If additional variants are subsequently discovered based on A1.1, they are named A1.1.1, A1.1.2, A1.1.3, etc. This system allows for the traceable classification of variant lineages and the relationship between original and derived forms. The amino acid variations and positions of all variants detected in this study as well as known variants for each of the 6 major milk proteins were shown in Supplementary Table S5-S10. These tables present the correspondence between established variant names and the novel variants with newly proposed nomenclature. The relationship between all variants were visualized using network plots presented in Figs. 2, 3, 4, 5, 6 and 7. The frequencies of variants across different breeds are presented in Supplementary Tables S11–S16. A summary of the known milk protein variants detected and those not identified in this study is presented in Table 2.

**Table 2:** Summary of known protein variants which are detected and not detected in this study. Variants in bold were detected in breeds not included in this study. Underlined variants were classified reported in literature as “rare”

Milk Protein	Known variants detected in current study	Known variants not-detected variants in current study
α_S1_-CN	B, C, D, I, J	A, E, F, H
β-CN	A1, A2, A3, B, C, F, I,J, L	B2, D, E, G, H1*,* H2, K
α_S2_-CN	A, B, D	C, E
κ-CN	A, B, C, D, E, H, G2, I	B2, F1, F2, G1, J, K
α-LA	A, B, E, F	C, D
β-LG	A, B, B1, C, D, W	E, F, G, H, I, J, K

### Overview of detected milk protein variants

Of the 121 detected milk protein variants, 35 corresponded to previously known variants and 86 were novel. The most common known variants included α_S1_-CN B and C, β-CN A1 and A2, α_S2_-CN A, κ-CN A and B, α-LA B, and β-LG A and B. Several less common or rare variants displayed clear breed-specific distributions. Variants such as α_S1_-CN D, β-CN A3, C, and I, κ-CN E, and β-LG C and D were detected exclusively in European taurine breeds. The α_S2_-CN D variant, although predominantly present in European taurine breeds, was also identified in a few African and East Asian populations. In contrast, a number of variants including α_S1_-CN I and J, α_S2_-CN B, β-CN J and L, κ-CN H, α-LA A, E, and F, and β-LG W occurred mainly in Indian zebu, African, and East Asian native breeds with indicine introgression history. α_S2_-CN B, κ-CN H, and α-LA E also occurred at low frequencies in a few European taurine breeds. The 86 newly identified milk protein variants showed diverse occurrence patterns. We highlighted three representative patterns: 1) relatively common variants detected with more than 10 occurrences; 2) geographical-specific variants restricted to particular populations; and 3) extremely rare variants with less than 4 occurrences met the setting quality criteria but were supported by read depth less than 6. Details of the newly identified variants in each representative occurrence pattern are provided in Supplementary Table S17.

### Frequencies of functional variants

**Fig. 8:**
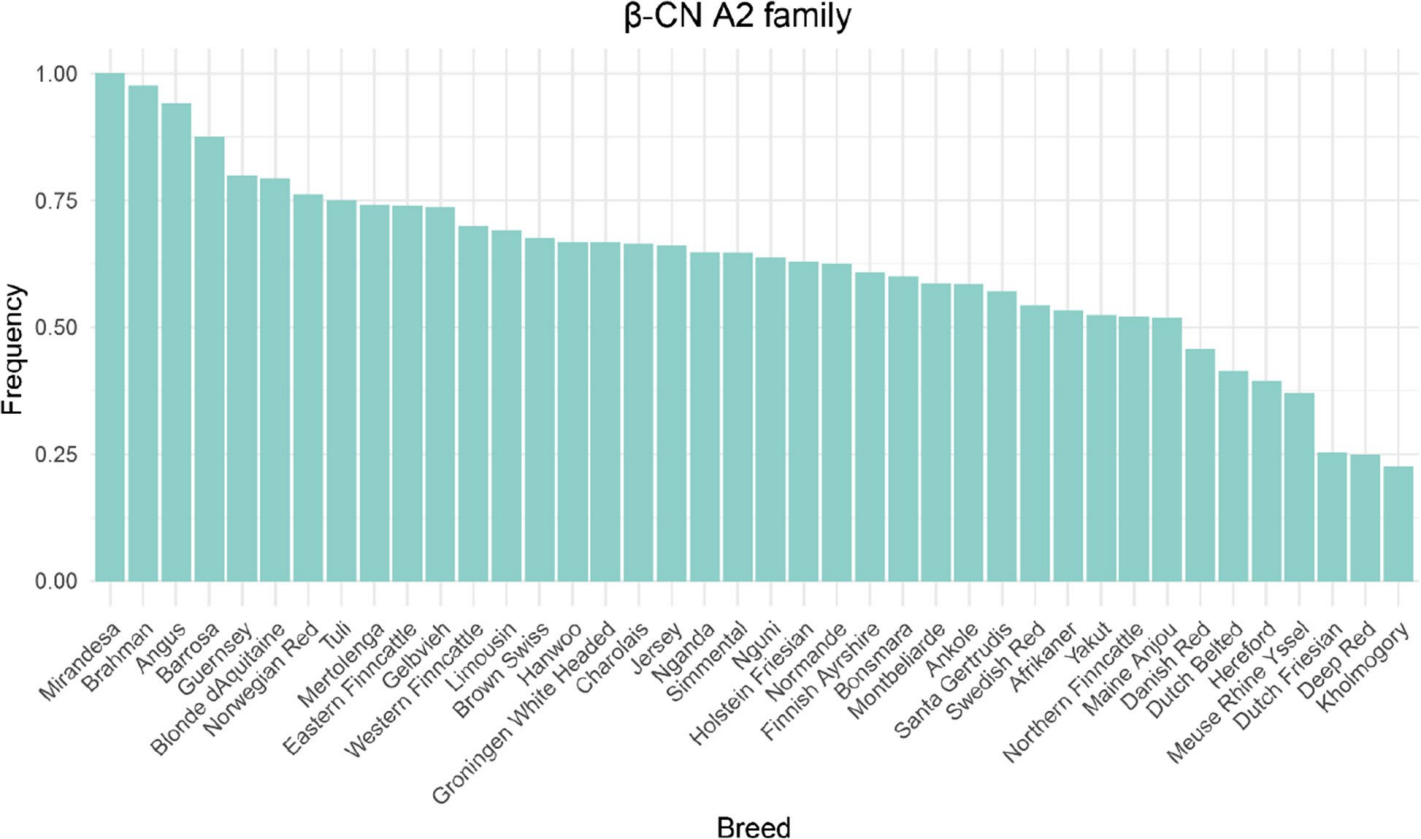
Frequency of β-CN A2 family variant in cattle breeds with at least 20 animals

**Fig. 9:**
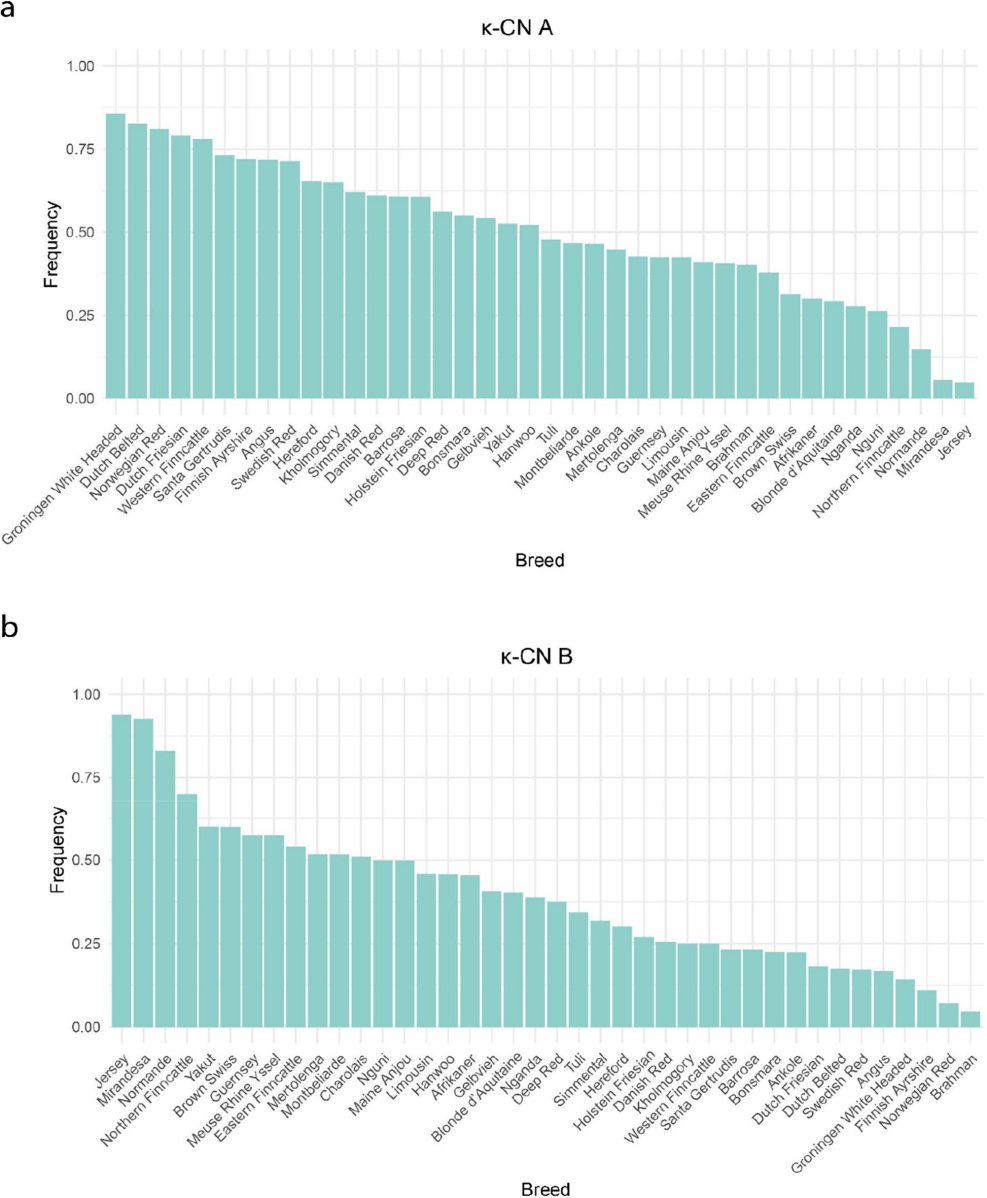
Frequency of κ-CN variant in cattle breeds with at least 20 animals. **a** Frequency of κ-CN A variant. **b** Frequency of κ-CN B variant

**Fig. 10:**
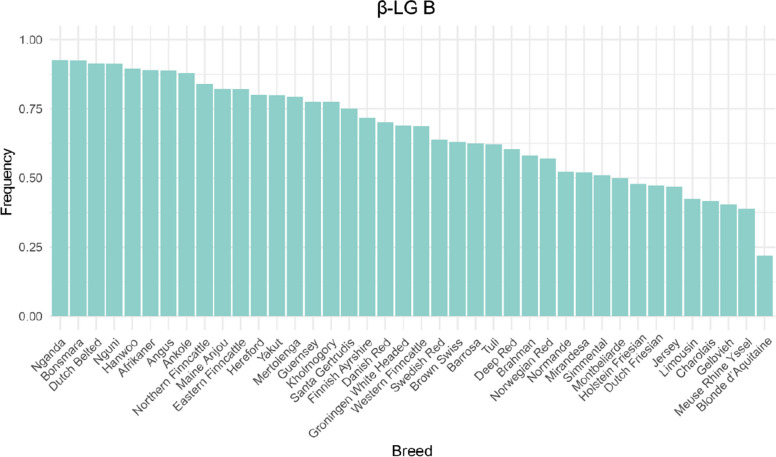
Frequency of β-LG B variant in cattle breeds with at least 20 animals

Frequencies of key functional variants including the β-CN A2 family referring to all β-CN variants carrying a proline residue at position 67, κ-CN A and B, and β-LG B variants in cattle breeds with at least 20 animals are illustrated in Figs. 8, 9a–b, and 10, respectively. β-CN A2 family showed intermediate to high frequencies in most breeds and appeared to be fixed in Mirandesa. It was also predominant in Brahman (0.976), Angus (0.942), and Barrosa (0.875). In contrast, four Dutch native breeds including Deep Red (0.25), Dutch Belted (0.413), Dutch Friesian (0.254) and Meuse Rhine Yssel (0.37) showed markedly lower A2 family frequencies, with the A1 variant being more common.

κ-CN A and B variants were both common variants and detected in all breeds. The B variant showed particularly high frequencies in Jersey (0.938), Mirandesa (0.926), and Northern Finncattle (0.700). The A variant was more frequent in Groningen White Headed (0.857), Dutch Belted (0.826), Norwegian Red (0.810), and Dutch Friesian (0.790) whereas the B variant occurred at lower frequencies. In other taurine breeds, frequencies of A and B variants were more balanced. β-LG B variant was predominated in most breeds, with particularly high frequencies in Nganda (0.926), Bonsmara (0.925), and Dutch Belted (0.913). In contrast, it was relatively rare in Blonde d’Aquitaine (0.219) and Meuse Rhine Yssel (0.398).

## Discussion

### DNA sequencing variants

In this study, we analyzed sequences of six milk protein genes as well as 2,000 bp upstream and downstream regions. The variations detected in the upstream and downstream regions may include functional regulatory elements such as promoters or enhancers that modulate gene expression. Several variants in the upstream region have been reported to influence transcriptional activity and gene expression, and consequently affect milk composition traits [7, 8, 21, 22].

In our dataset, the level of polymorphism within milk protein genes is higher than the average observed across bovine genes, where approximately 2.1% of base pairs are polymorphic [20]. We observed that 97.16% of DNA variants were located in the UTR, synonymous sites, and splicing regions and did not alter the amino acid sequences. This proportion is consistent with previous reports, which ranged from 94.2% to 98.9% [4, 18, 19] and lower compared with the genome-wide average of 98.43% [20]. Although such type of sequence polymorphisms do not result in amino acid changes of proteins, they have been shown to contribute to the genetic variance of milk production traits in mammary gland [23]. Moreover, variants within UTRs may cause the gain or loss of microRNA binding sites or transcription factor motifs, thereby leading to expression differences of the genetic protein variants [3, 4, 19]. Together, these results emphasize that genetic variants although silent at the protein level, can play a critical role in modulating gene expression and thereby contributing to functional diversity.

### Missense variant distribution

A total of 59 missense variants that change the amino acid sequence of the mature forms of six milk were detected. Variants located within predicted post-translational modification (PTM) sites may alter the phosphorylation or glycosylation levels, thereby influencing the technological properties of milk and dairy products [9, 24, 25]. Moreover, the distribution of missense variants highlights non-conserved and conserved regions across milk protein genes. We observe that the missense variants of *CSN3* gene almost exclusively located within the region coding for the hydrophilic κ-CN C-terminal (residues 113–169), which is part of the soluble casein macropeptide (CMP) after cleavage by chymosin between residues Phe^105^–Met^106^ [26, 27]. In contrast, other regions of *CSN3* are highly conserved with few sequence variations. In this study, only one variation was found in the region of *CSN3*, which codes for the para-κ-CN of κ-CN (residues 1–95). This region is the hydrophobic part that remains in the micelle after cleavage by chymosin. In an evolutionary study on κ-CN, Manguy and Shields noted that the length of para-κ-CN is relatively constrained, which might be due to its critical role in stabilizing the casein micelle internal structure [28]. However, evolutionary large insertions were tolerated in the CMP [28], which is the part of κ-CN that is exposed on the exterior of the micelle. These results indicated a higher conservation level of para-κ-CN compared to the CMP, which is consistent with the results of our study. Overall, the conserved regions of milk protein genes reflect strong structural and functional constraints, particularly in casein genes that play crucial roles in micelle assembly and stability [29].

### Known variants detection using WGS data

The frequencies of known variants across different breeds were largely consistent with those reported in earlier DNA-based studies, considering differences in sample size and geographic origin [18, 30–32]. Distinct geographic and ancestry-related distribution patterns were observed for several milk protein variants. Variants previously considered zebu-specific including α_S1_-CN I and J, β-CN J and L, α_S2_-CN B, κ-CN H, α-LA A, E, and F, and β-LG showed a wider distribution, particularly in Indian zebu, African and East Asian native breeds [4, 5, 33]. Moreover, α_S2_-CN B, κ-CN H and α-LA A, E were also detected in several southern European cattle. This distribution pattern is consistent with previous studies reporting the introgression of indicine ancestry into African, East Asian cattle and limited in Southern European cattle [34, 35].

Compared with previous studies that characterized protein variants using protein separation techniques, some difference were observed. For example, we detected the β-CN I variant which co-elutes with the A2 variant when analyzed by capillary zone electrophoresis [36]. Similar chromatographic limitations were reported for the discrimination between κ-CN A and E, α_S1_-CN B and C, and among different α_S2_-CN variants of A, B and D [37]. The use of DNA sequencing in the present study allowed for accurate discrimination of such variants. Moreover, we identified the κ-CN G2 and β-LG B1 variant at low frequencies in more than 30 breeds, including mainstream dairy, beef and native breeds. κ-CN G2 was originally identified in Pinzgauer cattle but has not been reported since [38]. The B1 variant has been detected only in the Beninese cattle using DNA sequencing data [4]. The inconsistent detection of κ-CN G2 and β-LG B1 variants in breeds in the current and previous studies is most likely due to limitations of the protein separation techniques that were used. Our study identified such variants effectively using WGS data and provide the precise frequencies among breeds.

### Undetected known variants

Some milk protein variants have been described in other studies but were not identified in the current study. The α_S1_-CN G variant caused by a large insertion in the noncoding region of *CSN1S1* gene resulted in lower amounts of α_S1_-casein protein in milk due to that reduces mRNA stability and transcription [39]. κ-CN AI variant is a new DNA variant of *CSN3* gene but results in a silent mutation at the amino acid sequence of κ-CN [40]. These two variants are not caused by amino acid changes and were therefore not considered in this study. α_S1_-CN A variant was not detected in this study because it is caused by a distal splice site variation [41]. However, the genetic variation responsible for α_S1_-CN A variant has not been experimentally validated yet. Thus appropriate milk samples are needed to clarify effects of potential splice site variants on the mature protein amino acid sequences [42].

Other variants appear to be specific to breeds or subspecies that were not represented in our analysis. Variants including α_S1_-CN H, α_S2_-CN E, κ-CN D, I, J, K were found to be specific for cattle breeds which were not included in this study [3]*.* Variants α_S1_-CN E, α_S2_-CN C, α-LA C, β-LG E, F and G were found to be specific for other *Bos* species such as *Bos grunniens* and *Bos javanicus*. Additional variants including α_S1_-CN F, β-CN D, E, H1 and H2, κ-CN F1 and F2 have been reported in other studies, but at low frequencies (~ 0.01) [43–45]. The α-LA D variant was detected in 1 of the 1,948 Dutch Holstein Friesian cows [6]. β-CN G variant was identified in 7 of 158 Holstein Friesian cows [46]. Taken together, these variants are only present in specific breeds or herds, which likely explains why these variants were not detected in the current study.

### Functional implication of known variants

Some variants that have been validated to be functional can be used in selective breeding in dairy sector due to their potential implication in human health. The β-CN A2 family variants, which have a proline residue at position 67, result in reduced release of β-casomorphin-7 (BCM-7), a peptide hypothesized to be associated with certain adverse health effects, although this remains controversial [47]. Regarding the manufacturing properties, κ-CN B is associated with improved milk rennet properties, increased cheese yield, and higher milk protein percentage [17, 48]. Moreover, milk with κ-CN B variant shows greater heat stability at natural pH [49]. κ-CN B is reported to be more resistant to gastric digestion compared with A variant [50]. β-LG B is associated with improved firmer curd and high dried weight cheese yield, and also show better foaming properties [49, 51]. In our dataset, the β-CN A2 family variants were fixed in Mirandesa and predominant in Brahman, and Angus. κ-CN B showed high frequencies in Jersey, Mirandesa, and Normande. The β-LG B variant was enriched in Nganda, Bonsmara, and Dutch Belted. The high frequencies distribution in breeds suggests that they could serve as valuable genetic resources for breeding programs aiming to improve specific processing properties of milk in the dairy sector. However, most Dutch native breeds displayed low frequencies of β-CN A2 family and κ-CN B variants. This may provide opportunities for breeders to increase the A2 milk production and cheese-making properties through selective breeding or genomic selection. In summary, the frequency distribution of functional variants across breeds provides valuable insight for designing breeding strategies aimed at improving the milk quality.

We observed a high frequency of the κ-casein A variant in both major commercial beef and dairy breeds. Among beef breeds, the frequency ranged from 0.425 in Limousin to 0.718 in Angus, with intermediate values in Charolais (0.427) and Hereford (0.635). In the main dairy breeds, the frequency was 0.606 in Holstein Friesian and 0.414 in Brown Swiss, but only 0.047 in Jersey. The E variant was a rare variant and occurred at low frequencies in both beef and dairy breeds. The comparable frequencies of these two variants between beef and dairy breeds indicate no clear evidence of differential selection, which contrasts with our initial hypothesis that stronger purifying selection might have acted against these variants in beef cattle, where milk composition directly influences calf growth and fitness. Our results suggest that these variants are not under strong functional constraint or that their effects are counterbalanced by other selective pressures across breeds. Remarkably, the κ-casein A variant is extremely rare in Jersey, reflecting long-term selection in favor of the κ-CN B allele, which improve milk quality and cheese-making properties [14, 52].

### Novel variants characterization

Novel milk protein variants can be the result of novel missense variants or recombination events between existing polymorphisms that result in new haplotypes. We detected 86 previously unreported variants across six milk proteins in this study. One reason why novel variants have not been detected previously might be the difficulty to separate the protein variants based on the analytical methods used before [15, 30, 37, 53–55]. Moreover, our large-scale data including diverse origin resulted in a higher probability to identify new variants. Our newly proposed hierarchical nomenclature system for the novel variants builds upon and extends the existing variant nomenclature providing a scalable and traceable framework to organize an increasing number of milk protein variants. This nomenclature approach may serve as a practical framework for broader application in future studies.

The novel variants which are relatively common and were detected across multiple breeds or with multiple occurrences are considered as high reliability. We observe that some novel variants were exclusively present in specific native breeds. This may be attributed to the limited number of studies conducted on these breeds. Because most of them have traditionally been raised for beef or draft rather than dairy production, genetic diversity in milk protein genes has remained largely unexplored. The reliability of novel variants depends heavily on the DNA sequencing quality as well as on the accuracy of haplotype reconstruction. We have applied strict criteria for filtering of the sequencing data which is identical to previous study [18] and included the missense variant needs to been detected in at least two animals. However, changes in the read depth threshold directly influence genotype calling. When we change the criteria of read depth more than 6, 8 of variants (α_S1_-CN B2, B2.1, B3, β-CN B2.1, B3, B4, β-LG E1, A3) were not present. This reflects that genotyping outcomes are sensitive to coverage stringency, and small changes in read depth thresholds can influence haplotype inference. Additionally, the accuracy of phasing by BEAGLE 5.4 depends mainly on the number of individuals, marker density, and relatedness [56]. As a result, genotypes were not 100% phased, and haplotype frequencies cannot be directly estimated by gene counting [57]. In this case, we applied EM algorithm implemented in the *haplo.stat* of R package to estimate haplotype likelihood frequencies, which resolving uncertainty in phase information and provides robust haplotype frequency estimates.

### Potential implication of novel variants

We observed that some novel variants were detected exclusively in indigenous breeds from Europe, India, Africa and East Asia, respectively, suggesting distinct genetic backgrounds between these populations [34, 58]. Although the physiological and functional significance of these native breed-specific variants remains unknown, they may represent the unique milk protein characteristic across native breeds that is absent from commercial populations [59, 60]. Characterizing these unique variants and conserving the native breeds that harbor them can help prevent irreversible loss. Therefore, these native breed-specific variants should be prioritized for follow-up characterization, particularly aiming at expanding milk protein variant resources and physiological investigation. Some newly identified variants may be relevant for milk functionality, because amino acid substitutions can influence PTMs or the structure of mature protein and thereby affect technological properties. Specifically, relative common novel variant κ-CN A5.2 and rare newly identified κ-CN variants including A4.1, B3.1.1, and H2.1.1 include a newly identified substitution (p.Ser149Phe) at phosphorylation site. This amino acid change may affect phosphorylation pattern of κ-CN and thus have potential effect on casein micelle formation [9]. Thus in the future, these functional variants could considered as prioritization to characterize and investigate biological or structural role, and further assess their potential suitability for the design or innovation of specific dairy products.

## Conclusion

This study provides a comprehensive overview of milk protein variants across global cattle breeds, identifying 121 distinct protein forms, including 35 known and 86 novel variants. The conserved region within the *CSN3* para-κ-casein domain implies functional importance in micelle formation and stability, whereas casein macropeptide region display higher tolerance for mutation. The frequencies distribution of functional variants across breeds expands our understanding of milk protein diversity and provides a valuable genomic resource for dairy breeding programs aiming at improving milk quality and properties. The application of large-scale WGS data enables the detection of numerous novel variants, providing foundation of conservation and dairy products innovation with specific biological and technological characteristics, and offering new perspectives for functional validation in future studies.

## Materials and methods

### Animals and sequencing data

The raw sequence data were derived from two sources. We first used 544 animals from LEAP-Agri project OPTIBOV (https://www.optibov.org/) including 10 native cattle breeds originating from 3 European countries (Finland, Netherlands, Portugal), 12 native cattle breeds from 3 African countries (Egypt, Uganda, South Africa) and 30 Holstein Friesian cattle from these 6 countries. Blood samples were collected and DNA was extracted from EDTA-blood using the GENTRA Blood kit (Qiagen N.V.) and processed according to the protocol previously described by previous study [58]. To be specific, DNA was extracted from EDTA-blood samples using the GENTRA Blood kit (Qiagen N.V.). The quality and quantity of DNA were assessed using a Qubit fluorometer (Qiagen N.V.). DNA-sequence libraires were prepared by using the DNA Library Prep Kit (Illumina Inc., USA) and paired-end 150 bp sequenced on the Illumina NovaSeq6000 platform (Illumina Inc., USA). In addition, the raw sequence variation data of 1000 Bull Genome Project Run 7.0 were used in this study [20]. Crossbred individuals were removed and only breeds with at least 2 animals were selected for the analyses, so that the final dataset contained 96 different breeds and 3,280 animals. By combining information from the OPTIBOV and the 1000 Bull Genome Project, a total of 3,824 animals from 113 breeds were included in this study. Detailed information about breeds and number of animals per breed can be found in Supplementary Table S1.

### Sequence data processing

Variants and genotypes of the OPTIBOV Project were called against reference genome assembly ARS-UCD1.2 using the haplotype-based method implemented in Freebayes [59]. Data of the 1000 Bull Genome Project, which was generated by aligning to the ARS-UCD1.2 reference genome, was used to filter for raw sequence variants [20]. Due to substantial variation in sequencing coverage across individuals, we applied filtering criteria based on thresholds established in a previous study [18]. SNP variants were only considered after aligning to the reference genome when at least three reads of the variant were detected. Moreover, SNPs with phred-scaled probability < 20, mapping quality < 20 and present in < 2 individuals were filtered out. Beagle 5.4 was used to infer haplotypes for genomic regions containing the 4 major casein genes on BTA6 and for the 2 major whey protein genes on BTA5 and BTA11 respectively using default settings [60].

### Functional annotation of DNA sequence variants

Sequence variants located within the 6 major milk protein genes *CSN1S1*, *CSN1S2*, *CSN2*, *CSN3*, *LALBA*, *PAEP* (Table 3) including 2,000 bp upstream and downstream were selected for analyses. The positions of the identified sequence variants were inferred considering the gene sequence positions in the ARS-UCD1.2 genome assembly [61]. Variants were classified as novel when not found in the dbSNP and European Variation Archive—EVA database [62]. All the variants were categorized into variant types based on their genomic locations (2,000 bp upstream, 5′-UTR, intron, synonymous, missense, 3′-UTR, 2,000 bp downstream) using the Ensembl Variant Effect Predictor v111 (VEP) [63]. Additionally, the functional consequences of missense variations on protein were predicted using the VEP. The VEP results from two VCF files (OPTIBOV and 1000 Bull Genome Project) were combined to subsequently calculate the frequency of each variant type. Allele frequencies of missense variants in different breeds were calculated using BCFtools [64].

**Table 3:** Reference gene sequences used to investigate polymorphisms in milk protein genes

Gene Name	Gene ID	Transcript ID	BTA^a^	Start (bp)	End (bp)	Strand^b^	Size (bp)	Exon number
*CSN1S1*	ENSBTAG00000007695	ENSBTAT00000010119.3	6	85,411,118	85,429,268	+	18,151	19
*CSN2*	ENSBTAG00000002632	ENSBTAT00000003409.6	6	85,449,164	85,457,744	−	8,581	9
*CSN1S2*	ENSBTAG00000005005	ENSBTAT00000006590.6	6	85,529,905	85,548,556	+	18,652	18
*CSN3*	ENSBTAG00000039787	ENSBTAT00000028685.5	6	85,645,854	85,658,926	+	13,073	5
*LALBA*	ENSBTAG00000005859	ENSBTAT00000007701.2	5	31,183,432	31,186,209	+	2,778	4
*PAEP*	ENSBTAG00000014678	ENSBTAT00000019538.6	11	103,255,824	103,260,873	+	5,049	7

^a^BTA*: Bos taurus* chromosomes

^b^+ = forward strand, − = reverse strand

### Milk protein variant identification

For each milk protein gene, haplotypes were constructed, as milk protein variants can be the result of combinations of two or more sequence variants in the coding regions. Haplotypes were constructed using the function *haplo.group* from R package *haplo.stats* with the default settings for the whole population and then within each breed using the EM algorithm [65]. DNA information obtained based on haplotypes were translated to amino acids and annotated to the milk protein variants following the existing nomenclature [3–6] by referring to the amino acid position in the mature proteins. Additionally, milk protein variants not found in literature are considered novel.

## Supplementary Information

Additional file 1: Supplementary Figure S1. Violin plot showing the distribution of sequencing read depth at genomic positions harboring missense variants in *CSN1S1* gene. Supplementary Figure S2. Violin plot showing the distribution of sequencing read depth at genomic positions harboring missense variants in *CSN2* gene. Supplementary Figure S3. Violin plot showing the distribution of sequencing read depth at genomic positions harboring missense variants in *CSN1S2* gene. Supplementary Figure S4. Violin plot showing the distribution of sequencing read depth at genomic positions harboring missense variants in *CSN3* gene. Supplementary Figure S5. Violin plot showing the distribution of sequencing read depth at genomic positions harboring missense variants in *LALBA* gene. Supplementary Figure S6. Violin plot showing the distribution of sequencing read depth at genomic positions harboring missense variants in *PAEP* gene.

Additional file 2: Supplementary Table S1. Origin of breeds or samples. Supplementary Table S2. List of the polymorphisms detected within six milk protein genes (*CSN1S1, CSN2, CSN1S2, CSN3, LALBA, LGB*) and their 2,000-bp upstream and downstream region. Supplementary Table S3. Number of variants (proportion in parentheses) found in the milk protein genes (*CSN1S1, CSN1S2, CSN2, CSN3, LALBA, PAEP*) and their 2,000-bp upstream and downstream region. Supplementary Table S4. Description (location, allele and ID) of the missense variations of the six milk protein genes (*CSN1S1, CSN2, CSN1S2, CSN3, LALBA, LGB*), their effects on the protein and allele frequencies in different breeds. Supplementary Table S5. Amino acid exchanges and position within the mature protein of α_S1_-CN variants in Bos genus. Variants in green sheet were known variants detected in this study. Variants in grey sheet were novel variants detected in this study. Supplementary Table S6. Amino acid exchanges and position within the mature protein of β-CN variants in Bos genus. Variants in green sheet were known variants detected in this study. Variants in grey sheet were novel variants detected in this study. Supplementary Table S7. Amino acid exchanges and position within the mature protein of α_S2_-CN variants in Bos genus. Variants in green sheet were known variants detected in this study. Variants in grey sheet were novel variants detected in this study. Supplementary Table S8. Amino acid exchanges and position within the mature protein of κ-CN variants in Bos genus. Variants in green sheet were known variants detected in this study. Variants in grey sheet were novel variants detected in this study Supplementary Table S9. Amino acid exchanges and position within the mature protein of α-LA variants in Bos genus. Variants in green sheet were known variants detected in this study. Variants in grey sheet were novel variants detected in this study. Supplementary Table S10. Amino acid exchanges and position within the mature protein of β-LG variants in Bos genus. Variants in green sheet were known variants detected in this study. Variants in grey sheet were novel variants detected in this study. Supplementary Table S11. The frequencies of α_S1_-CN variants in different breeds. Supplementary Table S12. The frequencies of β-CN variants in different breeds. Supplementary Table S13. The frequencies of α_S2_-CN variants in different breeds. Supplementary Table S14. The frequencies of κ-CN variants in different breeds. Supplementary Table S15. The frequencies of α-LA variants in different breeds. Supplementary Table S16. The frequencies of β-LG variants in different breeds. Supplementary Table S17. Novel milk protein variants grouped in different occurrence pattern.

## Data Availability

All genome sequences are publicly available and accessible. The raw VCF file of OPTIBOV project is available in European Variation Archive (EVA) under the project accession number PRJEB101802.

## Abbreviations

α_S__1_-CN: Alpha-S1-casein
β-CN: Beta-casein
α_S__2_-CN: Alpha-S2-casein
κ-CN: Kappa-casein
α-LA: Alpha-lactalbumin
β-LG: Beta-lactoglobulin
WGS: Whole-genome Sequencing
BTA: Bos Taurus Autosome
CMP: Casein macropeptide
PTM: Post Translational Modification
VEP: Variant Effect Predictor
EM: Expectation–Maximization
UTR: Untranslated Region
SIFT: Sorting Intolerant From Tolerant
BCM-7: Beta-casomorphine7
