# Supplementary material for: Global analysis of bovine milk protein variants using multi-breed DNA sequence data
Source: BMC Genomics. 2026 Apr 10;27:476. doi: 10.1186/s12864-026-12836-2 (PMC13182118; doi:10.1186/s12864-026-12836-2)
Supplement: Supplementary file 1 — Additional file 1: Supplementary Figure S1. Violin plot showing the distribution of sequencing read depth at genomic positions harboring missense variants in CSN1S1 gene. Supplementary Figure S2. Violin plot showing the distribution of sequencing read depth at genomic positions harboring missense variants in CSN2 gene. Supplementary Figure S3. Violin plot showing the distribution of sequencing read depth at genomic positions harboring missense variants in CSN1S2 gene. Supplementary Figure S4. Violin plot showing the distribution of sequencing read depth at genomic positions harboring missense variants in CSN3 gene. Supplementary Figure S5. Violin plot showing the distribution of sequencing read depth at genomic positions harboring missense variants in LALBA gene. Supplementary Figure S6. Violin plot showing the distribution of sequencing read depth at genomic positions harboring missense variants in PAEP gene. [file 12864_2026_12836_MOESM1_ESM.docx]

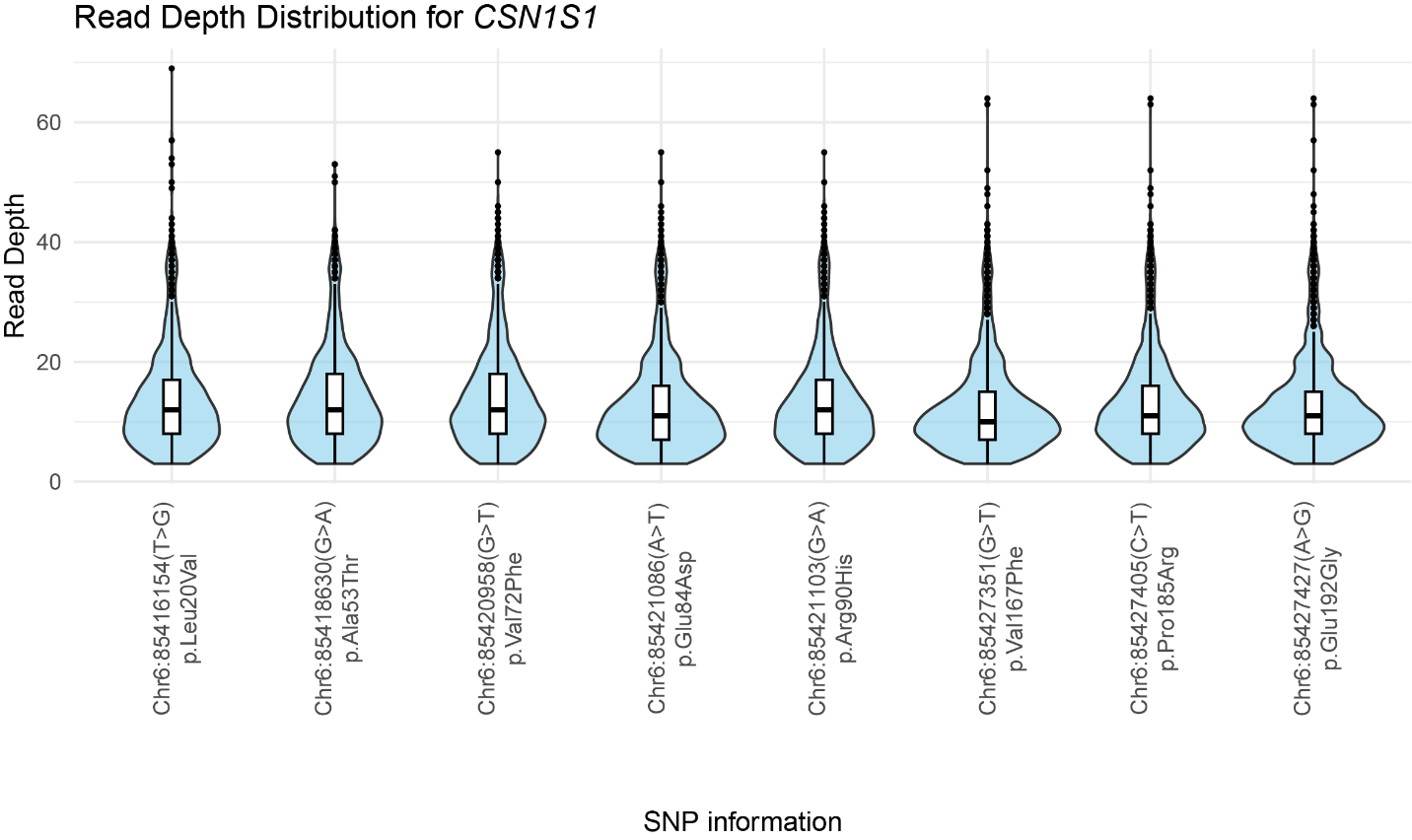


Supplementary Figure S1. Violin plot showing the distribution of sequencing read depth at genomic positions harboring missense variants in *CSN1S1* gene. The x-axis represents individual SNPs, labeled by their genomic positions, nucleotide substitutions (Reference allele> Alternative allele), and the corresponding amino acid changes. The white box within each violin represents the interquartile range of read depth, with the central black line indicating the median, and the outer shape showing the overall density distribution.


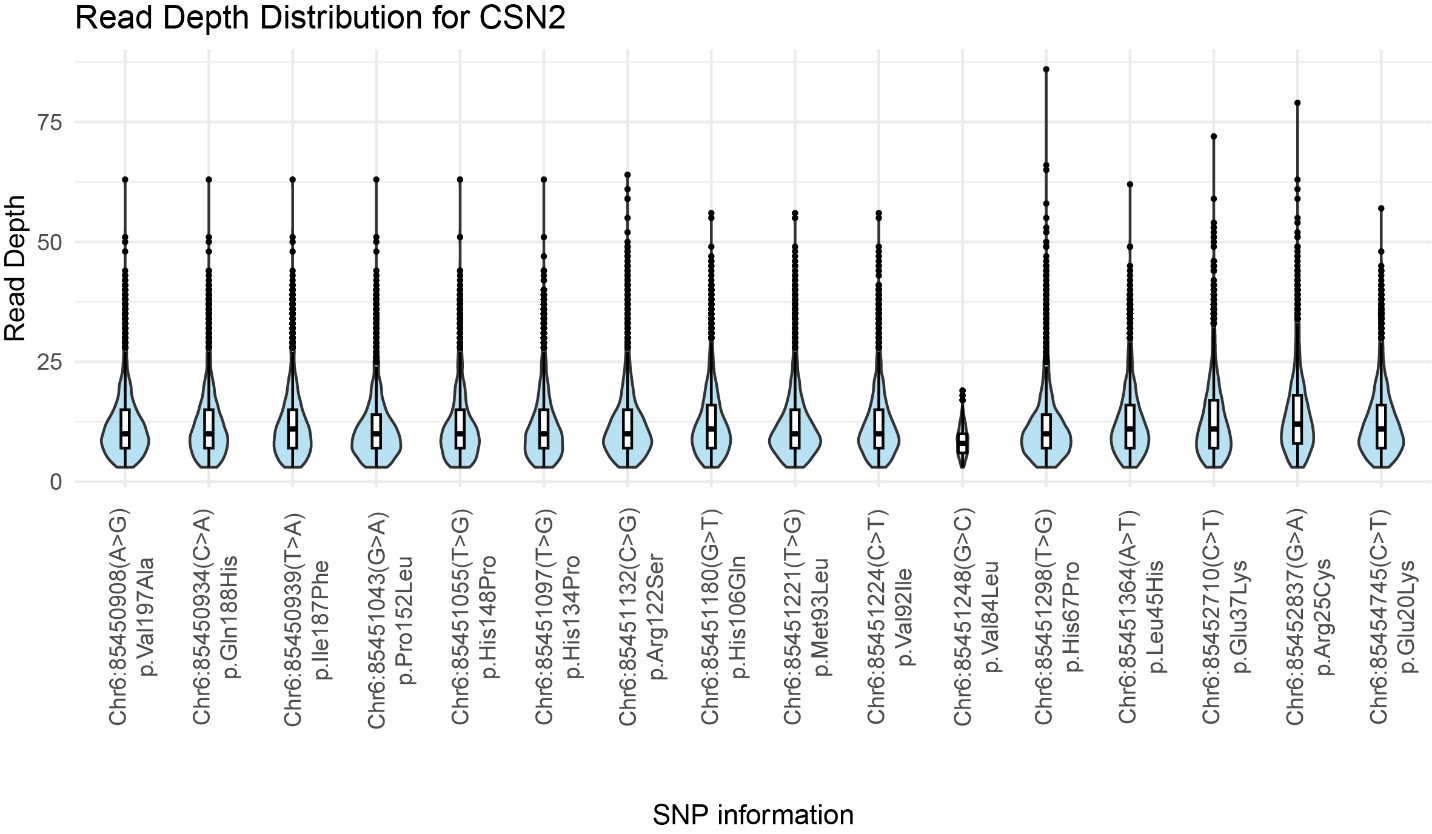


Supplementary Figure S2. Violin plot showing the distribution of sequencing read depth at genomic positions harboring missense variants in *CSN2* gene. The x-axis represents individual SNPs, labeled by their genomic positions, nucleotide substitutions (Reference allele> Alternative allele), and the corresponding amino acid changes. The white box within each violin represents the interquartile range of read depth, with the central black line indicating the median, and the outer shape showing the overall density distribution.


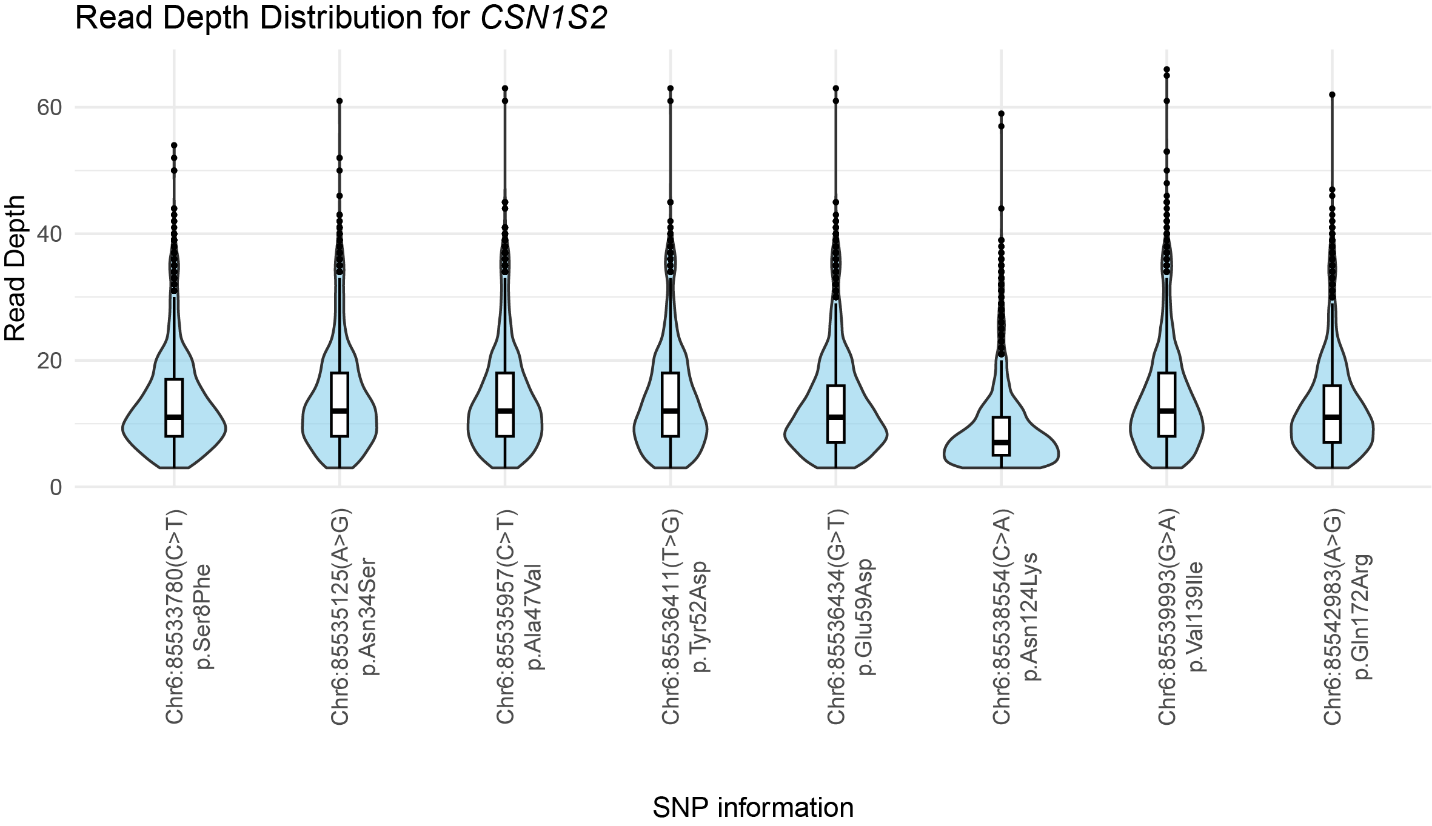


Supplementary Figure S3. Violin plot showing the distribution of sequencing read depth at genomic positions harboring missense variants in *CSN1S2* gene. The x-axis represents individual SNPs, labeled by their genomic positions, nucleotide substitutions (Reference allele> Alternative allele), and the corresponding amino acid changes. The white box within each violin represents the interquartile range of read depth, with the central black line indicating the median, and the outer shape showing the overall density distribution.


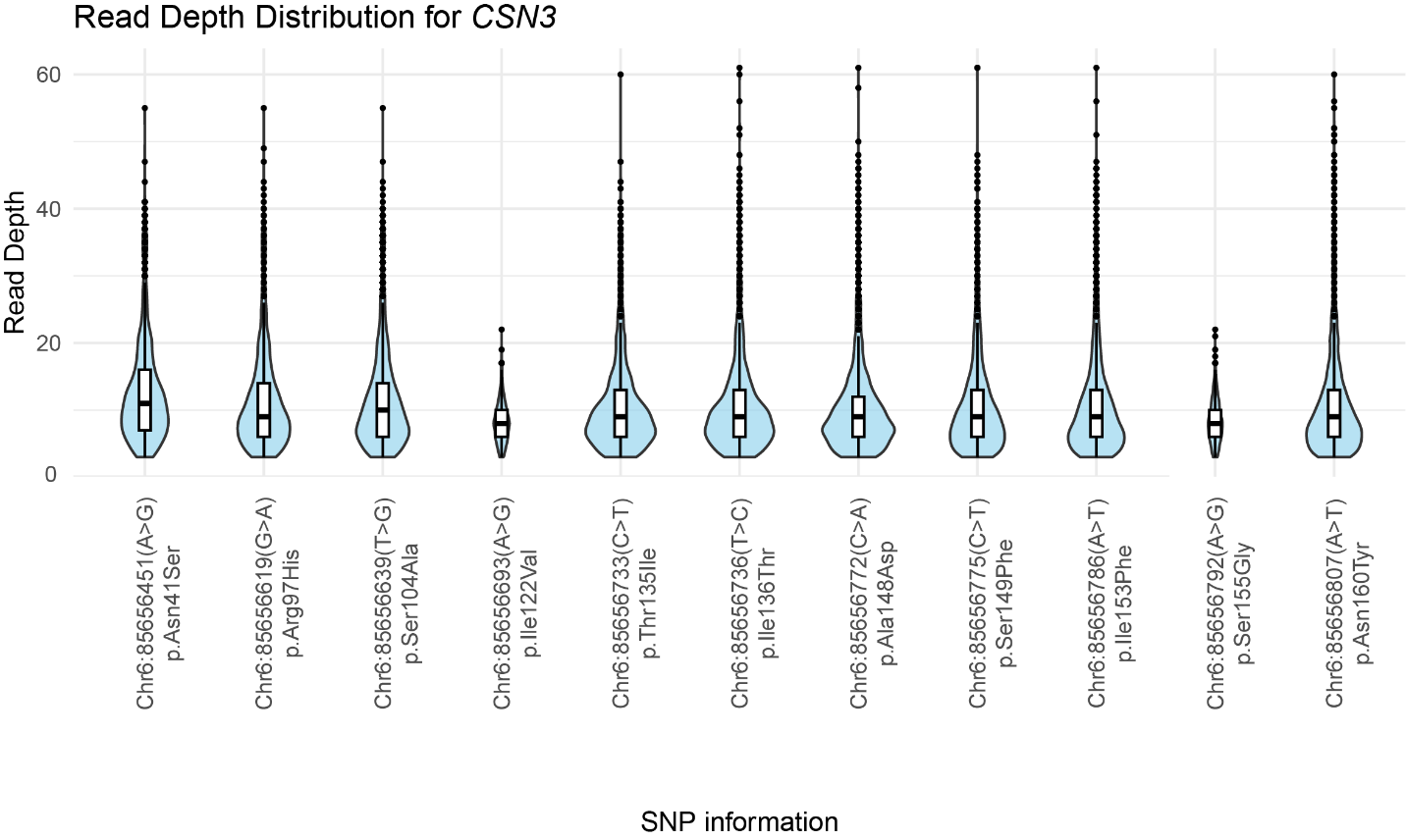


Supplementary Figure S4. Violin plot showing the distribution of sequencing read depth at genomic positions harboring missense variants in *CSN3* gene. The x-axis represents individual SNPs, labeled by their genomic positions, nucleotide substitutions (Reference allele> Alternative allele), and the corresponding amino acid changes. The white box within each violin represents the interquartile range of read depth, with the central black line indicating the median, and the outer shape showing the overall density distribution.


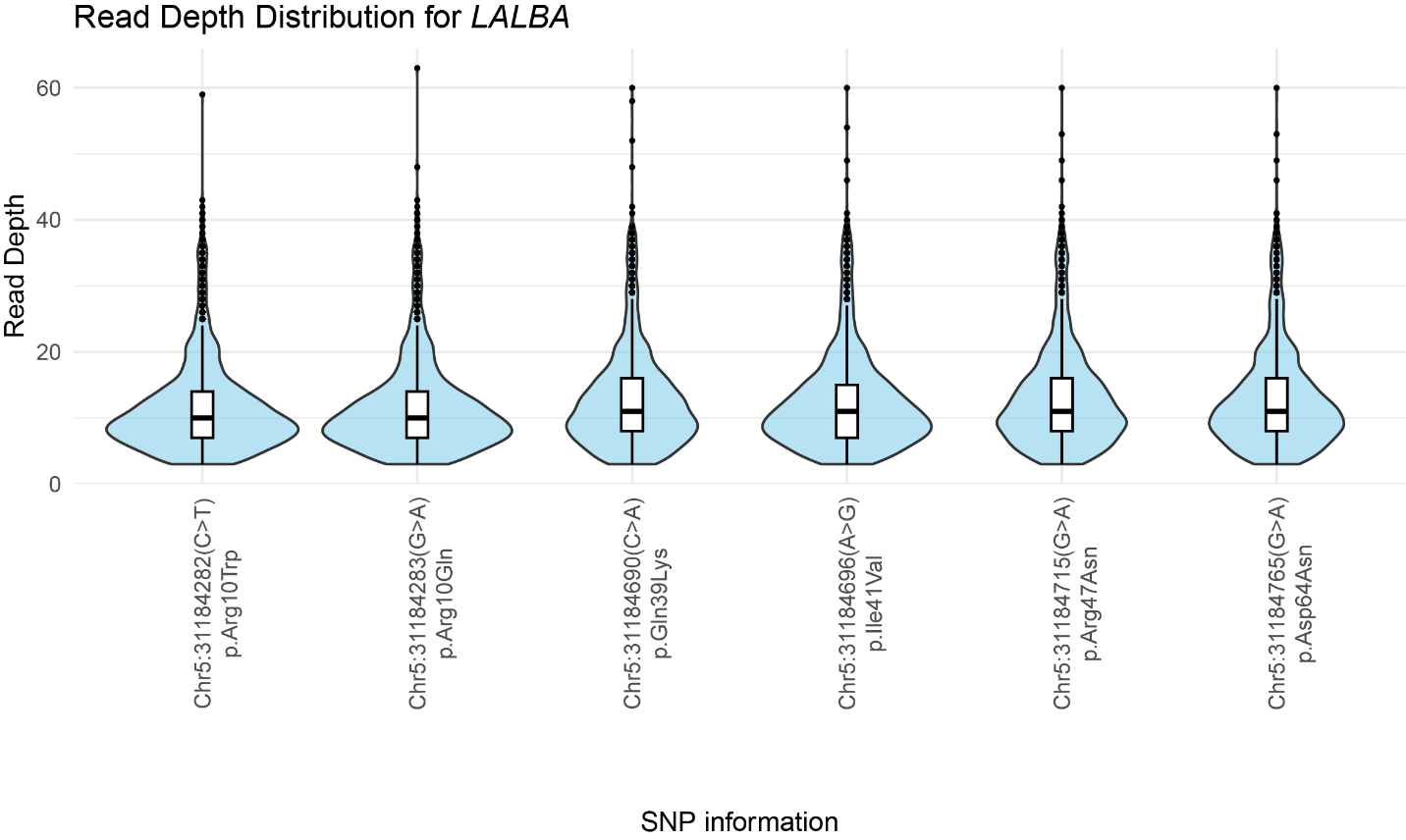


Supplementary Figure S5. Violin plot showing the distribution of sequencing read depth at genomic positions harboring missense variants in *LALBA* gene. The x-axis represents individual SNPs, labeled by their genomic positions, nucleotide substitutions (Reference allele> Alternative allele), and the corresponding amino acid changes. The white box within each violin represents the interquartile range of read depth, with the central black line indicating the median, and the outer shape showing the overall density distribution.


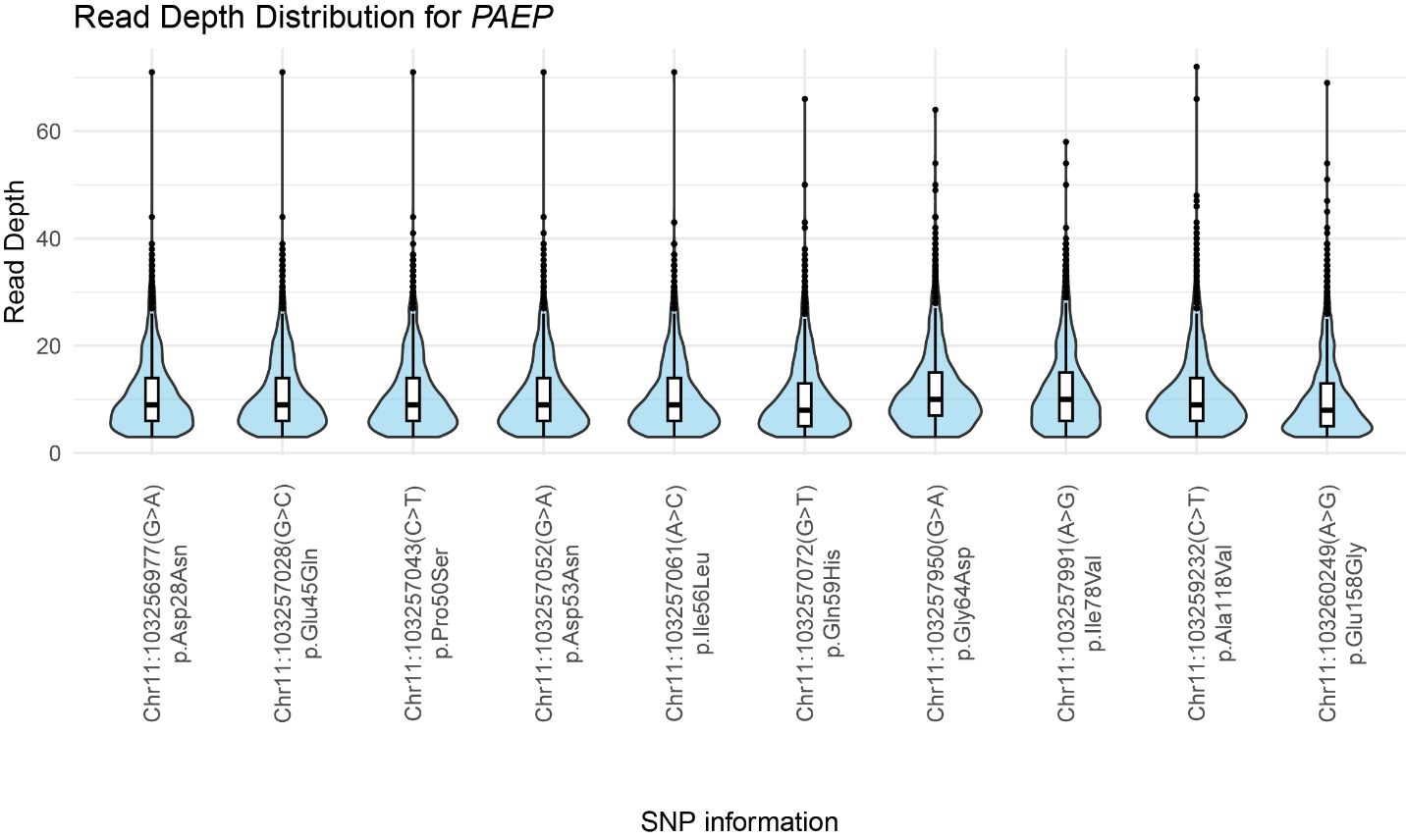


Supplementary Figure S6. Violin plot showing the distribution of sequencing read depth at genomic positions harboring missense variants in *PAEP* gene. The x-axis represents individual SNPs, labeled by their genomic positions, nucleotide substitutions (Reference allele> Alternative allele), and the corresponding amino acid changes. The white box within each violin represents the interquartile range of read depth, with the central black line indicating the median, and the outer shape showing the overall density distribution.
